# Clinical observations of EVO-ICL implantation and changes in corneal astigmatism using a modified technique

**DOI:** 10.1186/s12886-022-02603-8

**Published:** 2022-09-23

**Authors:** Ying Wang, Ruibo Yang, Yue Huang, Chen Zhang, Hui Liu, Zhe Jia, Shaozhen Zhao

**Affiliations:** Tianjin Key Laboratory of Retinal Functions and Diseases, Tianjin Branch of National Clinical Research Center for Ocular Disease, Eye Institute and School of Optometry, Tianjin Medical University Eye Hospital, Tianjin, 300384 China

**Keywords:** Implantable collamer lens, Modified technique, Intraocular pressure, Corneal endothelial, Corneal surgically induced astigmatism

## Abstract

**Background:**

Implantable collamer lens (ICL) surgery techniques are constantly progressing. The purpose of this study was to investigate the application effect of the modified technique and its impact on the change in corneal astigmatism in EVO-ICL surgery.

**Methods:**

The analysis of retrospective cohort data included 153 eyes of 81 patients with myopia from July 2018 to May 2020. An EVO-ICL was inserted by modified surgical skills, including a single 3.0 mm corneal incision and no ophthalmic viscosurgical device (OVD) before the insertion of the ICL (modified technique group: 41 cases, 80 eyes) and standard procedure (standard technique group: 40 cases, 73 eyes). Early postoperative intraocular pressure (IOP) was monitored at 2 and 24 h. IOP, corrected distance visual acuity (CDVA), uncorrected distance visual acuity (UDVA), vault, and anterior chamber depth (ACD) were measured 1, 6, and 12 months following the initial examination. The corneal endothelial cell density (ECD) was monitored at 6 and 12 months after the operation. Surgically induced astigmatism (SIA) in the total, anterior, and posterior corneal surfaces was analysed 1 month after the operation.

**Results:**

No serious complications were detected. The two groups had no difference in visual outcomes, ICL vaults, or ACD at any time point (*P* > 0.05). Two hours postoperatively, IOP was significantly lower in the modified technique group (16.22 ± 2.22 vs. 18.37 ± 1.92 mmHg, *P* < 0.05) than in the standard technique group. IOP decreased gradually after 24 h to preoperative levels. The postoperative IOP remained stable over a 12-month period. The ECD at 6 and 12 months was not significantly different between the groups (*P* > 0.05). SIA in the total, anterior, and posterior corneal surfaces were assumed to have no clinically meaningful differences between groups at one month after operation (*P* > 0.05).

**Conclusions:**

The modified technique is efficient and safe, producing comparable visual and structural outcomes without adversely affecting ECD, and reduces fluctuations in IOP at the early postoperative stages. The auxiliary incision in the standard technique does not increase corneal SIA, which is also a factor to consider for inexperienced surgeons.

## Background

The EVO implantable collamer lens (ICL) by the STAAR Surgical has been widely recognized as an efficient and safe treatment for individuals suffering from ametropia. As a result of extensive clinical practice based on standard surgical procedures, we simplified the standard surgical procedures and used a modified technique since July 2019. The modified technique involved a single corneal incision of 3.0 mm and no ophthalmic viscosurgical device (OVD) prior to ICL insertion.

The OVD can protect intraocular tissues as well as decrease fluctuations in intraocular pressure (IOP), which has been widely used during eye surgery [1]. However, the use of an OVD also has some disadvantages; it can obstruct the chamber angle and trabecular meshwork, resulting in an increase in IOP soon after surgery [2]. A drastically elevated IOP could permanently damage the endothelial cells and optic nerve. Thus, to avoid such complications, experienced surgeons use an OVD less frequently during ICL implantation. Our modified technique reduced the use of an OVD.

Furthermore, we analysed surgically induced corneal astigmatism due to changes in the incision from double to single. Even though modern refractive surgery rarely induces astigmatism due to the small incision size and absence of sutures to the wound, surgically induced astigmatism (SIA) is essential to further improve vision and patient satisfaction, particularly with toric ICL (TICL) implants.

The present study assessed the safety of the modified technique compared to the standard technique, specifically its impact on IOP and endothelial cell density (ECD), as well as the clinical effects on corneal SIA.

## Methods

### Patients

In this retrospective case series analysed, the patients evaluated were implanted with ICLs at Tianjin Medical University Eye Hospital between July 2018 and May 2020. A total of 81 participants (153 eyes) were investigated, consisting of 23 males (44 eyes) and 58 females (109 eyes). The inclusion criterion in the study was stable refraction power (less than 0.5 D increase in myopia every year for over two years), corrected distance visual acuity (CDVA) ≥ 10/20, the value of anterior chamber depth (ACD) had to be 2.80 mm or higher, ECD had to be more than 2000 cells/mm^2^, and the Pentacam HR (Oculus Instruments, Germany) quality specifications (QS) had to be “OK”.The participants with eye diseases (such as corneal diseases, ocular hypertensive, ocular trauma, uveitis, glaucoma, cataract, and retinal detachment) and systemic diseases (connective tissue disease, and diabetes mellitus.) affecting vision were excluded. All investigators followed the principles of the Helsinki Declaration 2008, and Tianjin Medical University Eye Hospital’s ethical committee approved the protocol (2022KY(L)-18).

### Data collection and image analysis

Before surgery, the patients were subjected to a comprehensive ophthalmic evaluation, including CDVA, uncorrected distance visual acuity (UDVA), slit-lamp microscopy, funduscopy, IOP (Canon, Japan), white-to-white (WTW), ECD (Topcon, Japan), and UBM (Quantel Medical, Clermont‐Ferrand, France). Central corneal thickness (CCT) and axial length (AL) were determined by LENSTAR (Haag-Streit, Switzerland). Measurements of ACD, ICL vault, and corneal astigmatism were performed by Pentacam HR eye examination. IOP was monitored 2 and 24 h postoperatively. Data for the UDVA, CDVA, IOP, ICL vault, and ACD were collected at 1, 6, and 12 months postoperatively. ECD was recorded at 6 and 12 months postoperatively. The SIA was quantitatively assessed at one month postoperatively. SIA was calculated using the ANSI method [3]. All examinations were completed by an experienced specialist.

### ICL operation procedure

The operation was conducted by the same senior surgeon (SZZ). Before the surgery began, an ICL was inserted into a STAAR Surgical injector cartridge. In the modified technique group, after one 3.0 mm corneal incision at the 11:00 position was made (right superotemporal and left superonasal), no OVD (Amvisc; Bausch & Lomb, Shandong, China) was used before inserting the ICL. After the ICL was inserted, an OVD was injected to help the ICL adjust into the posterior chamber. A cataract phacoemulsification machine with coaxial irrigation and aspiration (I/A) function was applied to clear the OVD. In the standard technique group, a 1.0 mm superior auxiliary incision was made, through which an appropriate OVD amount was injected into the anterior chamber, and a 3.0 mm main incision at the 11:00 was made at 90 degrees to the auxiliary incision. ICL implantation was performed through the main incision. After another OVD was administered over the ICL, the haptics tip was positioned on the ciliary sulcus via the auxiliary incision, and the intraoperative anterior chamber OVD was removed via I/A. As soon as the surgery was completed, the incisions were closed with a balanced salt solution. Following the surgery, we prescribed combined treatment with steroidal and antibacterial eye drops, as well as artificial tears.

### Statistical analysis

The statistical evaluation was conducted by SPSS 25.0 (IBM Corporation, USA). We adopted the Kolmogorov‒Smirnov test to validate the normal data distribution. In addition, we used nonparametric and parametric tests for comparisons of continuous variables based on the specific distribution of data. The independent *t* test or Mann–Whitney *U* test was used for continuous variables analysis, while the Fisher’s exact test or the chi-square test were implemented to compare categorical variables and conduct evaluations of the intergroup differences. The IOP, ACD, vault and ECD were evaluated using the multivariate analysis of variance (MANOVA) for repeated measures, with treatment modality being the intergroup factor and time being the intragroup factor. Tukey’s post hoc multiple comparison tests were used for pairwise comparisons. Hotelling’s T-squared test was performed to compare the centroid values of corneal SIA between the two groups. Any differences at *P* < 0.05 were considered statistically significant.

## Results

In the present study, we included a total of 81 participants (153 eyes). The demographics and baseline preoperative indicators are listed in Table 1. In the intergroup comparison, no significant differences were identified regarding age, sex, UDVA, CDVA, manifest spherical equivalent, corneal astigmatism, IOP, AL, ACD, WTW, CCT, or ECD (*P*>0.05).

**Table 1:** Baseline demographic indicators of the study population

Parameters	Modified Technique Group (*N* = 80)		Standard Technique Group(*N* = 73)		*P Value*
	MEAN ± SD	RANGE	MEAN ± SD	RANGE	
Eye (n)	80		73		
Patients (n)	41		40		
Sex (Female:Male)	58:22		51:22		0.72
Age (year)	26.83 ± 6.90	18,46	27.90 ± 6.78	18,44	0.48
Spherical refraction (D)	-9.52 ± 2.83	-5.25, -18.00	-9.01 ± 2.43	-4.25, -14.00	0.24
Cylinder refraction (D)	-1.32 ± 0.87	-3.25,0	-1.34 ± 0.88	-3.50,0	0.85
Manifest spherical equivalent (D)	-10.18 ± 2.74	-5.25, -19.25	-9.68 ± 2.63	-4.50, -15.50	0.26
Total corneal astigmatism					
Magnitude (D)	1.46 ± 0.85	0.20, 4.0	1.53 ± 0.93	0.30,4.2	0.63
Meridian (°)	89.74 ± 17.31	9.50,156.0	91.15 ± 14.58	49.60,161.0	0.59
Anterior corneal astigmatism					
Magnitude (D)	1.47 ± 0.83	0.30,4.00	1.56 ± 0.88	0.30,3.90	0.53
Meridian (°)	90.21 ± 16.27	17.30,135.5	92.06 ± 14.04	54.40,164.9	0.45
Posterior corneal astigmatism					
Magnitude (D)	0.44 ± 0.14	0.20,0.90	0.42 ± 0.17	0.10,0.90	0.54
Meridian (°)	90.08 ± 11.23	11.00,106.00	90.98 ± 12.30	14.10,117.70	0.64
UDVA (log MAR)	1.44 ± 0.29	0.70,2.00	1.37 ± 0.26	0.70,2.00	0.11
CDVA (log MAR)	0.02 ± 0.04	0.00,0.20	0.02 ± 0.05	-0.10,0.20	0.75
IOP (mmHg)	14.81 ± 2.54	10.30,20.50	14.55 ± 2.54	9.40,19.30	0.54
AL (mm)	27.51 ± 1.54	24.60,31.88	27.14 ± 1.29	24.16,30.68	0.11
ACD (mm)	3.21 ± 0.19	2.81,3.60	3.26 ± 0.26	2.80,3.81	0.21
WTW (mm)	11.57 ± 0.35	10.76,12.62	11.49 ± 0.34	10.80,12.37	0.17
CCT (µm)	516.8 ± 32.45	431.00,633.00	513.2 ± 30.51	457.00,591.00	0.49
ECD (cells/mm^2^)	3064 ± 257.6	2515,3868	3001 ± 309.8	2380,3717	0.17

### Visual outcomes

Throughout the entire follow-up period, no patients’ postoperative CDVAs declined. Both groups’ mean postoperative CDVAs were better than or equal to their mean preoperative CDVAs, and no significant between-group difference was observed during the follow-up research (*P* > 0.05). We observed statistically significant improvements in both groups’ postoperative UDVAs (*P* < 0.05); however, we detected no statistical between-group difference during the follow-up durations.

### IOP, ACD, and Vault

The postoperative IOP was significantly higher at 2 h postoperation than before the operation (*P* < 0.05) in both groups. The IOP gradually returned to preoperative levels, and there was no difference before and 24 h afterward the operation (*P* > 0. 05). No significant within-group difference in IOP was noted afterward, and the IOP remained stable at 1, 6, and 12 months of follow-up. Compared with the standard technique group, the modified technique group’s IOP was significantly lower at 2 h (16.22 ± 2.22 vs. 18.37 ± 1.92 mmHg, *P* < 0.05) postoperatively. On the other follow-up visits, IOP did not differ statistically between the two groups.

**Fig. 1:**
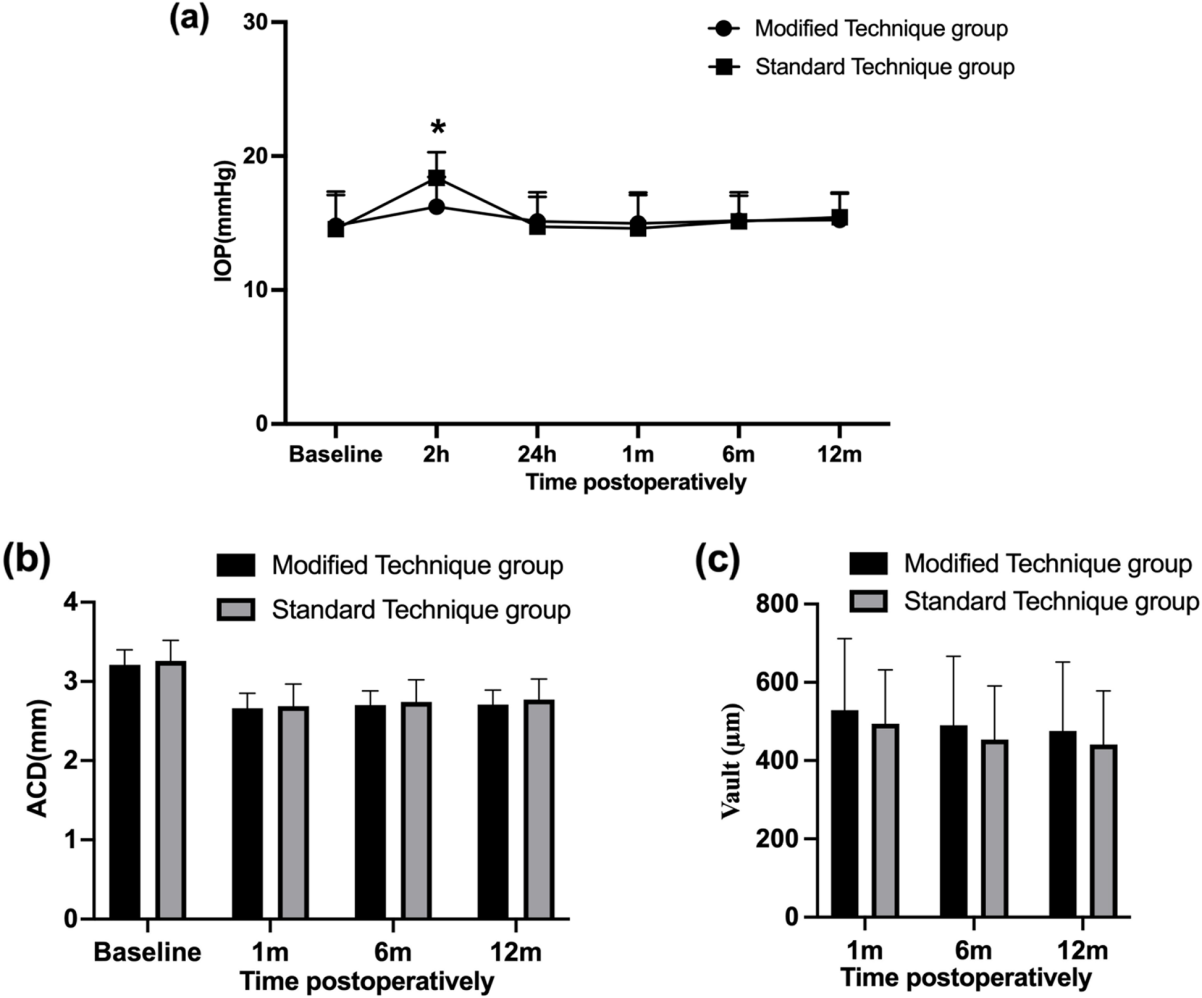
Mean IOP (**a**), ACD (**b**), and vault (**c**) of the modified technique group and the standard technique group at each follow-up different time points. * *P* < 0.05, the error bar indicates the standard deviation

The postoperative ACD was significantly lower than the preoperative value at 1 month after surgery (*P* < 0.05) and remained stable afterward in both groups. Additionally, we established no significant group differences in the values of ACD and ICL vault at the 1-, 6-, and 12-month postoperative follow-up durations (*P* > 0.05). The IOP, ACD, and ICL vault postoperatively are shown in Fig. 1.

### ECD

Both the modified and standard technique groups experienced a gradual decrease in ECD after surgery, but we detected no significant differences between them at 6 months (3025.0 ± 253.0 vs. 2962.0 ± 308.7 cells/mm^2^, *P* > 0.05) and 12 months (3015.0 ± 253.1vs. 2951.5 ± 308.5 cells/mm^2^, *P* > 0.05) postoperatively.

Additionally, we established no significant differences between groups in their values of the ECD loss rates at 6 months (1.27% vs. 1.29%, *P* > 0.05) and 12 months (1.60% vs. 1.67%, *P* > 0.05) postsurgery follow-up durations.

### SIA

We found no significant between-group difference in the mean absolute SIA of the total, anterior, and posterior corneas one month after surgery(*P* > 0.05) (Table 2).

**Table 2:** Mean absolute SIA of the total, anterior, and posterior corneas at one month after operation between the two groups

Parameters	Modified Technique Group(*N* = 80)		Standard Technique Group(*N* = 73)		*P Value*
	MEAN ± SD	RANGE	MEAN ± SD	RANGE	
Total corneal SIA (D)	0.60 ± 0.30	0.10,1.65	0.64 ± 0.30	0.17,1.58	0.45
Anterior corneal SIA (D)	0.60 ± 0.26	0.14,1.38	0.62 ± 0.25	0.10,1.36	0.79
Posterior corneal SIA (D)	0.15 ± 0.09	0.0,0.40	0.14 ± 0.09	0.0,0.40	0.52

**Fig. 2:**
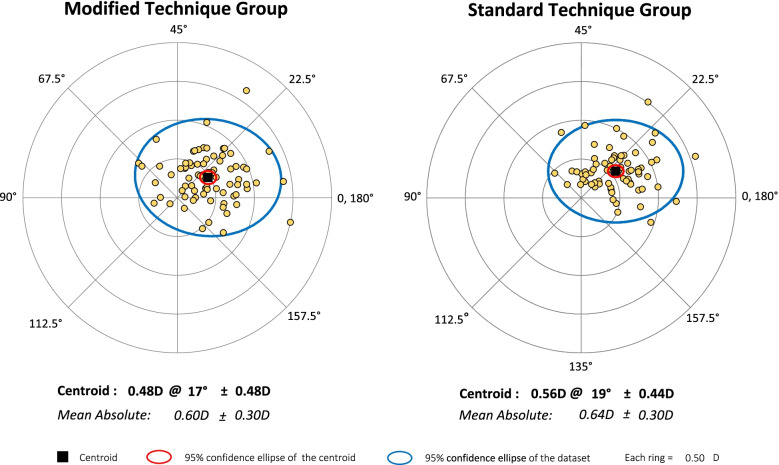
The SIA of the total cornea of the modified technique group (centroid: 0.48 × 17° ± 0.48 D) and the standard technique group (centroid: 0.56 × 19° ± 0.44 D) one month after surgery is shown in a double-angle plot. No significant difference was found in SIA between the two groups in centroid analysis (*P* > 0.05)

**Fig. 3:**
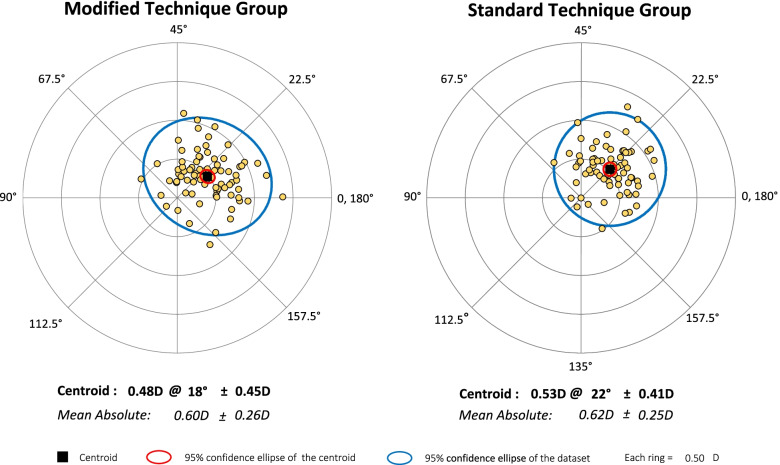
The SIA of the anterior cornea of the modified technique group (centroid: 0.48 × 18° ± 0.45 D) and the standard technique group (centroid: 0.53 × 22° ± 0.41 D) one month after surgery is shown in a double-angle plot. No significant difference was found in SIA between the two groups in centroid analysis (*P* > 0.05)

Figures 2 and 3 show the distribution of SIA in the total and anterior corneas. The distance from the original point of each spot equals the magnitude of astigmatism, and the angle equals the double astigmatism meridian. The double-angle plots were obtained with the tool available on the ASCRS website [4].

## Discussion

EVO-ICL implantation has achieved good effects in clinical applications in recent years [5–8]. EVO-ICL is fitted with a central hole to circulate the aqueous humour more naturally, reducing the need for peripheral iridotomies. Our results align with the relevant research results, suggesting that the modified technique is an effective and safe treatment for myopia and astigmatism [9, 10].

Postoperative elevation of IOP after ICL implantation is one of the problems of great clinical concern. The cause of high IOP postoperatively has been attributed to several factors, such as an OVD residue, angle closure and pupillary block by high vault. However, the elevation of IOP occurring shortly after the ICL implantation is most likely due to OVD retention [11–13], which is worthy of attention. Thorough removal of the OVD can take several minutes, and sometimes prove challenging, especially behind the ICL. When using the standard technique, the OVD may become stuck behind the ICL. A common problem with OVD is completely removing it behind the ICL, whereas the modified technique avoids such a problem. With the modified technique, the procedure could simplify the OVD removal and ensure that less is left in the posterior chamber. Elevated IOP is possible at an early postoperative stage. In previous studies [9, 14], IOP tended to peak at 2 h after surgery. Therefore, an IOP measurement was taken two hours after surgery. In our study, all participants’ IOP increased 2 h after surgery, with a lower increase in the modified technique group, which was analysed to be related to the use of less OVD and the concentration of OVD on the anterior surface of ICL that was easy to remove or discharge. IOP recovered to preoperative levels in both groups 24 h after surgery, indicating that the spike in IOP was temporary and might have been overlooked. In most cases, elevated IOP following ICL implantation is transient and does not require medical intervention, but in some cases, antiglaucoma medication or surgery may be required [15–17]. Anterior chamber drainage via incision is an effective method for the early postoperative treatment of high IOP. By releasing a small amount of aqueous hydrate, IOP can be reduced, and the residual OVD in the anterior chamber can be discharged through the incision as it drains. ACD and IOP tend to vary much. Attention was paid to the amount of anterior chamber fluid discharged from the incision. When possible, the liquid was discharged through an auxiliary corneal incision for enhanced safety.

A primary objective of the use of OVDs is crucial to maintain anterior chamber stabilization and protect intraocular tissue from surgical instruments and ICL during surgery [18]. The loss of ECD caused by ICL implantation occurs in the early postoperative stage due to injury to the corneal endothelium during the intraoperative operation. During the first 12 months after ICL implantation, ECD loss is most severe, and the rate of ECD loss typically slows down and stabilizes over time after 12 months postoperatively despite physiological ECD loss [19–22]. Although the corneal endothelium was less protected by the modified technique, the ECD was not different between groups due to the short surgical time, the well-maintained anterior chamber, and no mechanical injury to the corneal endothelium. These findings further confirm the feasibility and safety of the modified OVD reduction technique for experienced surgeons.

The modified technique could simplify the surgical process, reduce the OVD complications, decrease IOP fluctuations in the early postoperative period, and cause no additional damage to the endothelium compared to the standard technique. Some scholars have also adopted ICL surgery without OVD and have achieved good postoperative results [23–26].

Researchers have found that the ICL vault tends to decrease with time. There is a possibility of a decline in the vault as a result of eye movement, rotation of the ICL, haptic fixation location changes, and crystalline lens thickening with age [27, 28]. Alfonso et al. [29] showed that a monthly decline larger than 20 µm occurred in the vaults for six months postoperatively. This decrease was reduced to approximately 2 μm a month during the 36-month observation period. In our study, we established a continuous vault value decline over time, but the vaults of the ICLs remained within a normal range in all patients.

In the standard technique group, an additional auxiliary side incision of 1.0 mm was made. We should be aware that corneal incisions may increase the SIA of the cornea. The present study indicates that the SIA of both groups was not significantly different after surgery. ICL implants may induce astigmatism of approximately 0.5 D, which is not large but more than negligible for candidates seeking the greatest amount of refractive error correction. We consider that this information on the SIA of the cornea may prove helpful for refractive surgeons in planning procedures as well as for manufacturers of ICLs to enhance visual outcomes after ICL implantation. Most studies analysed the changes in astigmatism using the absolute value of the power of the cylinder. The mean absolute SIA is calculated from the astigmatism magnitude, without considering the astigmatism direction. While astigmatism consists of magnitude and axis parameters, its actual changing effect should be viewed as a vector. The magnitude and direction of astigmatism determine the centroid SIA, which is potentially helpful to assess SIA trends in surgery. Hence, the mean absolute and centroid SIAs were adopted to determine how astigmatism varies among patients treated with the different surgical techniques. A comparison of the mean absolute SIA and centroid SIA between the two groups did not reveal any differences. Therefore, for less skilled or inexperienced surgeons, there is no need to pursue a single incision. Auxiliary incisions reduce the number of instruments in and out of the main incisions, thereby improving anterior chamber stability, placing the haptics, and adjusting the position of the ICL. Additionally, auxiliary incisions can facilitate postoperative drainage. This increases surgery safety and decreases the risks of intraoperative and postoperative complications.

## Conclusions

The modified technique of implanting ICLs has been found to be satisfactory in terms of safety and efficacy, according to the present study. In comparison to the standard technique, the modified technique resulted in no adverse effects on ECD, decreased IOP fluctuations, and simplified operating procedures. SIA was not exacerbated by the auxiliary incision. Therefore, the modified technique may appeal to experienced surgeons, while two incisions are also a good alternative for inexperienced surgeons. In this study, there were some limitations since it was a retrospective cohort investigation. Hence, additional long-term studies are necessary to confirm our current results.

## Data Availability

The datasets generated and/or analysed during the current study are not publicly available due the protection of the rights and interests of patients by the Ethics Committee of Tianjin Medical University Eye Hospital but are available from the corresponding author on reasonable request.

## Abbreviations

ICL: Implantable collamer lens
IOP: Intraocular pressure
UDVA: Uncorrected distance visual acuity
CDVA: Corrected distance visual acuity
AL: Axial length
WTW: White-to-white
ACD: Anterior chamber depth
CCT: Central corneal thickness
ECD: Endothelial cell density
SIA: Surgically induced astigmatism
